# Closed Loop Direct Adaptive Inverse Control for Linear Plants

**DOI:** 10.1155/2014/658497

**Published:** 2014-01-19

**Authors:** Muhammad Amir Shafiq, Muhammad Shafiq, Nisar Ahmed

**Affiliations:** ^1^Faculty of Electronics Engineering, GIKI, Topi, Pakistan; ^2^Sultan Qaboos University, Muscat, Oman

## Abstract

In direct adaptive inverse control (DAIC), parameters of the controller are estimated directly in the feed-forward loop. In this paper, we propose a closed loop direct adaptive inverse control (CDAIC) scheme which improves tracking, error convergence, and disturbance rejection properties of DAIC. CDAIC is applicable to stable or stabilized, minimum or nonminimum phase linear plants. CDAIC and DAIC are compared using computer simulations for disturbance free and disturbed discrete type nonminimum phase linear plants. CDAIC shows better results compared to DAIC in terms of mean square tracking error and disturbance rejection.

## 1. Introduction

Adaptive control over the last five decades has emerged as one of the well-established discipline; see Astrom and Wittenmark [1], Gang and Rogelio [2], and Sastry and Bodson [3]. In adaptive inverse control (AIC), parameters of the inverse are obtained using direct or direct methods; refer to Widrow and Walach [4], Widrow and Bilello [5], Widrow and Plett [6], Plett [7], Shafiq [8], and M. Shafiq and M. A. Shafiq [9]. AIC has attracted the interest of many researchers for many years due to its computationally less expensive and robust tracking characteristics; see Widrow and Walach [4], Widrow and Bilello [5], and Yin et al. [10]. Majority of AIC schemes are developed for stable or stabilized plant and have been applied successfully to numerous practical problems such as temperature control of a heating process, speed control of a dc motor, control of kiln, control of nonlinear ship maneuvering, real time blood pressure control, and noise cancelation; see Shafiq [8], Dias and Mota [11], Du et al. [12], and Widrow and Walach [4]. All physical systems in the real world are inherently nonlinear in nature, but we linearize those plants around certain point and range to obtain linear behavior. If plant is unstable, then it is assumed that it was stabilized using any known control law before applying AIC scheme; refer to Widrow and Walach [4] and Ogata [13]. In this paper, we will discuss tracking schemes for discrete time stable or stabilized linear plants only.

Discrete type plants for which one or more zeros lie outside the unit circle are called nonminimum phase plants; see [14]. Nonminimum phase plant poses some controller design problems such as extra phase lag, step response in negative direction because their inverse is unstable; refer to Ogata [13]. Numerous techniques have been developed for control of minimum and nonminimum phase plants; refer to Widrow and Walach [4], Widrow and Bilello [5], Widrow and Plett [6], Plett [7], Shafiq [8], M. Shafiq and M. A. Shafiq [9], Bai and Dasgupta [15], and Wang and Chen [16]. In DAIC schemes inverse is designed based on identified plant; see Plett [7] and Shafiq et al. [9]. DAIC alleviates the adhocism in adaptive loop by directly estimating the inverse of the plant in feed-forward loop. Adaptive inverse control of linear and nonlinear systems using dynamic neural networks is presented in Plett [7]. In Plett [7], controller is designed based on identified plant. Similarly, DAIC scheme based on identification of the nonlinear autoregressive model with exogenous inputs (NARX) is presented in Yin et al. [10]. Direct and indirect model based control for nonlinear single input single output (SISO) plant using artificial neural networks are discussed in Wang and Chen [16]. DAIC based on neural network has also been successfully applied for controlling kiln; see Dias and Mota [11]. In this paper, we propose a closed loop direct adaptive inverse control technique based on normalized least mean square (NLMS) for controlling linear plants. CDAIC can be used for tracking of stable or stabilized, minimum or nonminimum phase linear plants.

The rest of this paper is organized as follows.  Section 2 presents problem statement. Details of the DAIC scheme are given in Section 3. Design of proposed scheme (CDAIC) is discussed in Section 4. Simulation results are given in Section 5 and finally conclusion is given in Section 6.

## 2. Problem Statement

Let us consider *P*(*q* ^−1^) is a discrete time stable or stabilized linear plant. Let *P*(*q* ^−1^) be given by

(1) $$\begin{array}{c} P\left(q^{-1}\right)=q^{-d}\frac{B\left(q^{-1}\right)}{A\left(q^{-1}\right)},\\ A\left(q^{-1}\right)=1+a_{1}q^{-1}+a_{2}q^{-2}+\cdots +a_{n}q^{-n},\\ B\left(q^{-1}\right)=b_{0}+b_{1}q^{-1}+b_{2}q^{-2}+\cdots +b_{m}q^{-m}, \end{array}$$

where *q* ^−1^ is a back shift operator defined as *q* ^−1^ *y*(*k*) = *y*(*k* − 1), *k* is positive integer that represents time instant, and *d* is a positive integer; it represents delay of the plant. We assume that *n* and *m* are unknown positive integers and *n* ≥ *m*. *A*(*q* ^−1^) and *B*(*q* ^−1^) are relatively coprime polynomials. We also assume that plant may be nonminimum phase; that is, inverse of plant is unstable. Let *r*(*k*), *y*_*d*_(*k*), and *y*(*k*) be the reference input, desired output, and plant output, respectively. Further, it is assumed that parameters of the plant are unknown or slowly time varying compared to the adaptation algorithm. The objective is to design a controller such that *y*(*k*) tracks *y*_*d*_(*k*); that is,

(2) $$\begin{array}{c} \underset{k\to \infty }{\lim}{\left(e_{\text{ref}}\left(k\right)\right)}^{2}=\underset{k\to \infty }{\lim}{\left(y_{d}\left(k\right)-y\left(k\right)\right)}^{2}=\epsilon , \end{array}$$

where *y*_*d*_(*k*) = *r*(*k* − *L*), *L* is a positive integer that represents known delay. *e*_ref_ (*k*) is error at instant *k* and *ϵ* is arbitrarily small positive real number in neighborhood of zero.

## 3. Direct Adaptive Inverse Control

**Figure 1:**
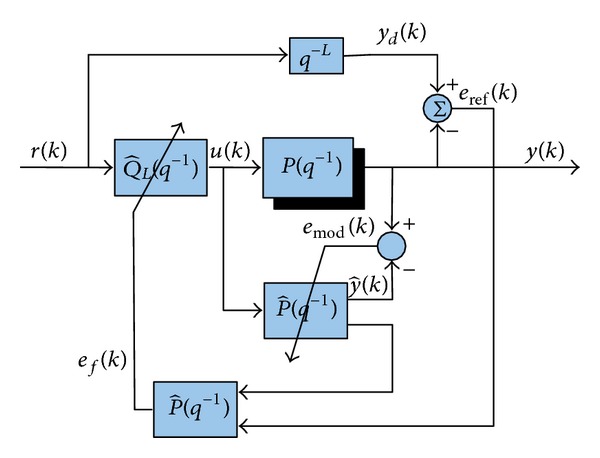
Direct adaptive inverse control.

DAIC scheme for controlling discrete time linear plants proposed in M. Shafiq and M. A. Shafiq [9] is shown in Figure 1. In DAIC, approximate inverse system ${\hat{Q}}_{L}(q^{-1})$ is directly estimated in feed-forward loop and control input *u*(*k*) is synthesized using

(3) $$\begin{array}{c} u\left(k\right)={\hat{Q}}_{L}\left(q^{-1}\right)r\left(k\right). \end{array}$$

In DAIC, first of all an approximate model of plant $\hat{P}(q^{-1})$ is estimated. Then *e*_*f*_(*k*) is obtained by back-propagating reference error *e*_ref_ (*k*) through estimated plant model $\hat{P}(q^{-1})$. Finally, *e*_*f*_(*k*) is used to adapt the weights of adaptive inverse controller. NLMS is used to estimate weights of plant model $\hat{P}(q^{-1})$ and adaptive inverse controller ${\hat{Q}}_{L}(q^{-1})$. Weight update equation for controller is given by

(4) θ(k+1)={θ(k)if  ψ(k)ψT(k)=0,θ(k)+μ1ef(k)ψ(k)ψ(k)ψT(k)if  ψ(k)ψT(k)≠0,

where *θ*(*k*) is parameter vector for ${\hat{Q}}_{L}(q^{-1})$. *μ*_1_ is learning rate and 0 ≤ *μ*_1_ ≤ 1. *ψ*(*k*) is regression vector defined as

(5) $$\begin{array}{c} \psi \left(k\right)=\left[r\left(k\right),r\left(k-1\right),\ldots ,r\left(k-N\right)\right], \end{array}$$

where *N* + 1 are number of controller parameters. DAIC alleviates the adhocism of adaptive loop by directly estimating the controller in feed-forward loop. Since plant model is identified first, DAIC is less sensitive to plant uncertainties and variations. DAIC depend on perfect estimation of plant model. Any nonlinearities or error in estimating correct plant model could degrade the performance of DAIC.

## 4. Design of CDAIC

**Figure 2:**
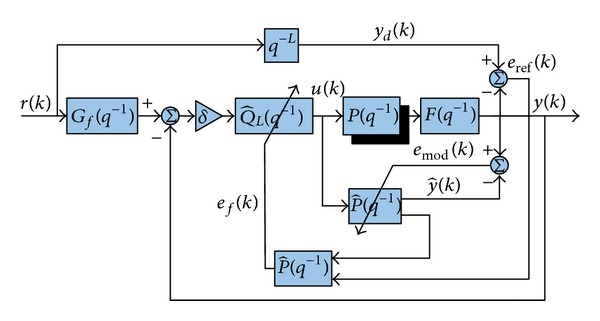
Closed loop direct adaptive inverse control.

We propose CDAIC structure shown in Figure 2. To the best of our survey, CDAIC scheme depicted in Figure 2 has not been reported in the literature. In this structure, feedback is used to improve the performance of DAIC. That is why we call it CDAIC. Control input to plant is synthesized such that plant tracks the desired input *y*_*d*_(*k*). Control input *u*(*k*) is given by

(6) $$\begin{array}{c} u\left(k\right)={\hat{Q}}_{L}\left(q^{-1}\right)\left(\Gamma \left(q\right)r\left(k\right)-\delta y\left(k\right)\right), \end{array}$$

where *y*(*k*) is the feedback from the plant. Γ(*q*) is FIR filter given by Γ(*q*) = 1 + *δq* ^−(*l*+*d*)^. *l* is a positive integer and *δ* is any positive real number such that 0 < *δ* < 1. *δ* also makes sure that control input remains bounded for bounded input and system does not become unstable. Steady state error is minimized using negative feedback. Mean square error (MSE) between desired output and plant output for nonminimum phase plants can be made small by incorporating the delay *q* ^−*L*^. ${\hat{Q}}_{L}(q^{-1})$ is used as feed-forward controller for *P*(*q* ^−1^). Since plant and its inverse are in cascade, they collectively form a transfer function which satisfies

(7) $$\begin{array}{c} q^{-(l+d)}{\hat{Q}}_{L}\left(q^{-1}\right)P\left(q^{-1}\right)F\left(q^{-1}\right)\approx q^{-L}. \end{array}$$

**Proof**

We assume that ${\hat{Q}}_{L}(q^{-1})$ is an approximate inverse of *P*(*q* ^−1^)*F*(*q* ^−1^); that is,

(8) $$\begin{array}{c} {\hat{Q}}_{L}\left(q^{-1}\right)P\left(q^{-1}\right)F\left(q^{-1}\right)\approx q^{-(l+d)}, \end{array}$$

therefore transfer function of inner closed loop is obtained as

(9) $$\begin{array}{c} G_{i}\left(q^{-1}\right)\approx \frac{\delta q^{-(l+d)}}{1+\delta q^{-(l+d)}}. \end{array}$$

It is clear that −1 < *δ* < 1 assures the stability of closed loop. The filter *G*_*f*_(*q* ^−1^) = (1 + *δq* ^−(*l*+*d*)^)/*δ* is incorporated in series with *G*_*i*_(*q* ^−1^). Now, the overall closed loop transfer function *G*_*c*_(*q* ^−1^) is given by

(10) Gc(q−1)=Gf(q−1)·Gi(q−1)≈1+δq−(l+d)δ·δq−(l+d)1+δq−(l+d)=q−(l+d)≈q−L.

**Remark 1 s**

Choosing 0 < *δ* < 1 makes *G*_*i*_(*q* ^−1^) a fast low pass filter. This property filters out the noise from the adaptive loop which insures smooth estimation of ${\hat{Q}}_{L}(q^{-1})$ parameters.

**Remark 2 s**

Main cause of oscillations in the parameters of the adaptive inverse controller is the noisy plant signal. The low pass filter behavior of the inner closed loop reduces oscillations in the parameter estimation and ultimately the plant output becomes smooth. The overall closed loop system becomes less sensitive to abrupt changes which enhance the robustness in the signal tracking.

**Remark 3 s**

Small positive values of *δ* reduces the open loop gain. This property improves the robustness in the closed loop stability.

*q* ^−*L*^ is generally kept small for minimum phase and large for nonminimum phase plants. Moreover, as the adaptive FIR filters are inherently stable, the controller will remain stable. In CDAIC,

(11) $$\begin{array}{c} \underset{k\to \infty }{\lim}{\left(e_{\text{ref}}\left(k\right)\right)}^{2}⟶0, \end{array}$$

provided 0 ≤ *μ*_2_ ≤ 1, 0 < *δ* < 1, and

(12) $$\begin{array}{c} \underset{k\to \infty }{\lim}{\left(e_{\mathrm{mod}}\left(k\right)\right)}^{2}⟶0. \end{array}$$

In order to use a noise free plant output, a first-order low pass butterworth filter *F*(*q* ^−1^) is used which follows the plant as shown in the Figure 2. Weight update equation for CDAIC controller is given by

(13) ω(k+1)={ω(k)if  φ(k)φT(k)=0,ω(k)+μ2ef(k)φ(k)φ(k)φT(k)if  φ(k)φT(k)≠0,

where *ω*(*k*) is parameter vector for CDAIC controller ${\hat{Q}}_{L}(q^{-1})$. *μ*_2_ is learning rate and 0 ≤ *μ*_2_ ≤ 1. *φ*(*k*) is regression vector defined as

(14) $$\begin{array}{c} \varphi \left(k\right)=\left[v\left(k\right),v\left(k-1\right),\ldots ,v\left(k-N\right)\right], \end{array}$$

where *N* + 1 are number of controller parameters and *v*(*k*) is given by

(15) $$\begin{array}{c} v\left(k\right)=\Gamma \left(q\right)r\left(k\right)-\delta y\left(k\right). \end{array}$$

## 5. Simulation Results

Computer simulations of CDAIC and DAIC scheme are presented to show effectiveness of CDAIC. Two linear nonminimum phase systems are chosen, one without disturbance and other with disturbance.

**Example 1**

A disturbance free discrete time nonminimum phase linear plant is chosen having

(16) $$\begin{array}{c} y\left(k\right)=q^{-1}\frac{1+2.5q^{-1}+3q^{-2}}{1+0.1q^{-1}+0.2q^{-2}+0.2q^{-3}+0.3q^{-4}}u\left(k\right). \end{array}$$

**Figure 3:**
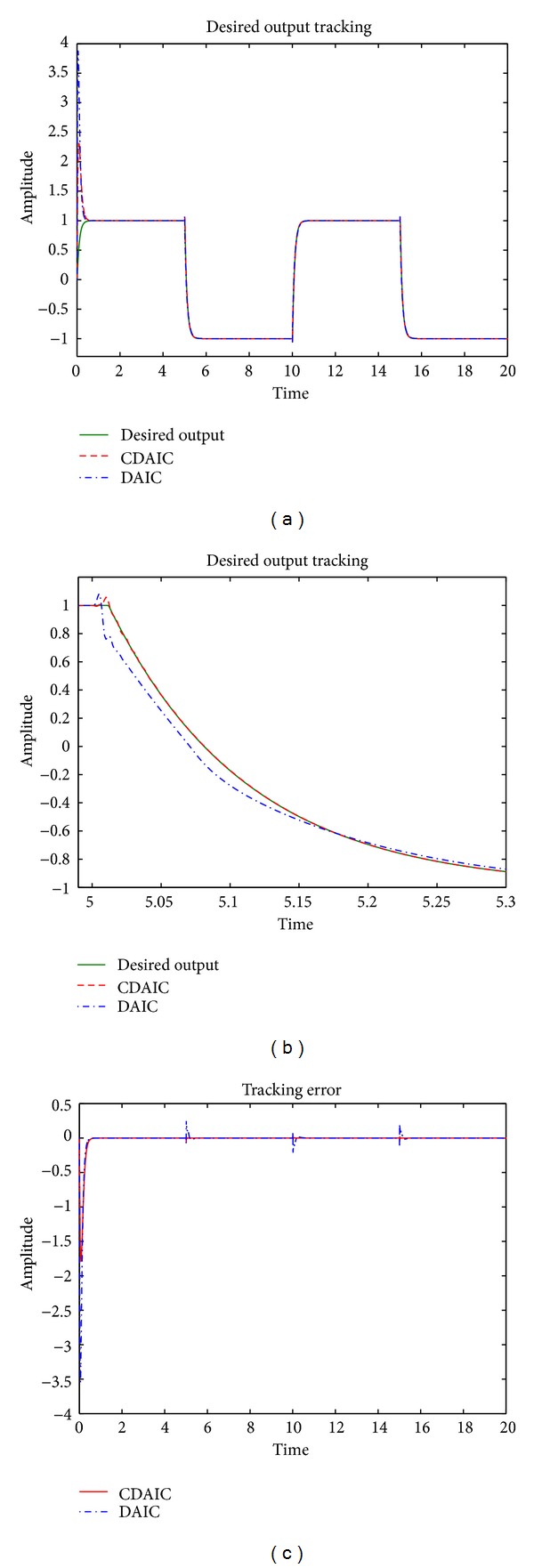
Example 1: simulation results: (a) tracking desired output; (b) tracking desired output (zoomed preview); (c) tracking error.

**Figure 4:**
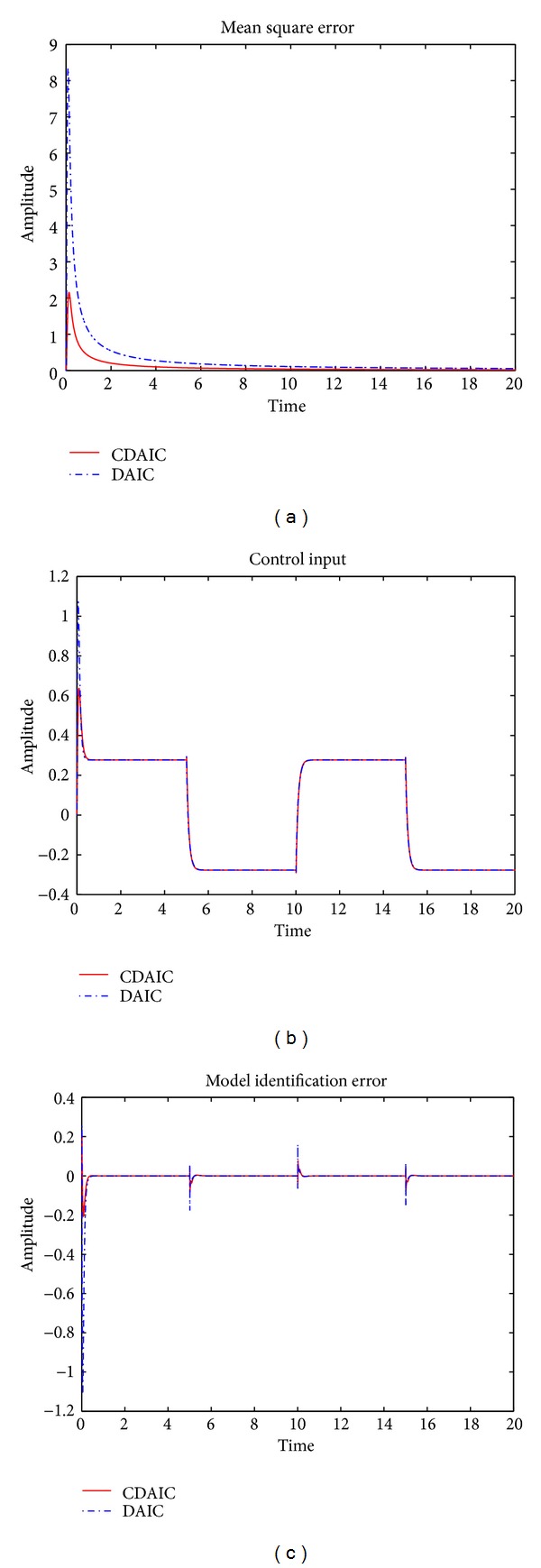
Example 1: simulation results: (a) mean square error; (b) control input; (c) model identification error.

This is a stable nonminimum phase plant having zeros at −1.2500 ± 1.1990*i*, poles at 0.4516 ± 0.6519*i* and −0.5016 ± 0.4749*i*. In this example, the learning rate of both CDAIC and DAIC is chosen as 0.001 for controller and 0.01 for plant. Also *δ* is chosen as 0.1 for CDIAC. Order of ${\hat{Q}}_{L}(q^{-1})$ for both CDAIC and DAIC is chosen as 10. Sampling time is chosen as 0.001 sec. Simulation results are depicted in Figures 3 and 4.

Desired output tracking is shown in Figures 3(a) and 3(b). Plant output in CDAIC has less overshoot and converges to desired output quickly compared to DAIC. Tracking error is shown in Figure 3(c). Tracking error has less amplitude and converges to zero faster in CDAIC compared to DAIC. Also CDAIC has low error at variations in desired output such as at instant 5 sec and 10 sec. MSE for CDAIC and DAIC is shown in Figure 4(a). MSE is less for CDAIC compared to DAIC. Control input is also shown in Figure 4(b). Control input in CDAIC is synthesized such that it converges faster and gives better tracking compared to DAIC. Model identification error *e*_mod_(*k*) is also shown in Figure 4(c). Model identification error in CDAIC converges quickly to zero compared to DAIC even for same leaning rate of plant model approximation.

**Example 2**

A disturbance *n*(*k*) is added to discrete time nonminimum phase linear plant. Plant output can now be written as

(17) $$\begin{array}{c} y\left(k\right)=q^{-1}\frac{B\left(q^{-1}\right)}{A\left(q^{-1}\right)}\left(u\left(k\right)+n\left(k\right)\right), \end{array}$$

where

(18) $$\begin{array}{c} B\left(q^{-1}\right)=1+5q^{-1}+8q^{-2},\\ A\left(q^{-1}\right)=1+0.5q^{-1}+0.1q^{-2}+0.3q^{-3}+0.7q^{-4}. \end{array}$$

**Figure 5:**
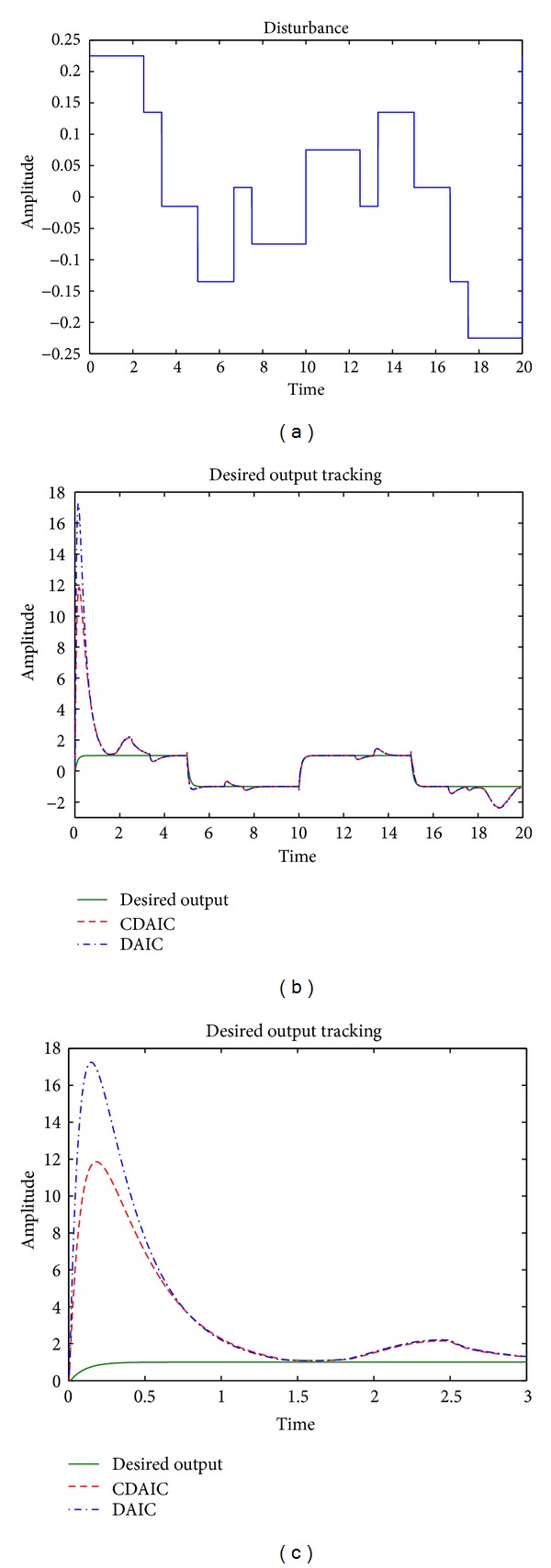
Example 2: simulation results: (a) disturbance; (b) tracking desired output; (c) tracking desired output (zoomed preview of first 3 sec).

This is a stable nonminimum phase plant having zeros at −2.5 ± 1.3229*i*, poles at −0.7650 ± 0.5470*i* and 0.5150 ± 0.7254*i*. In this example, the learning rate of both CDAIC and DAIC is chosen as 0.0001 for controller and 0.001 for plant. Also *δ* is chosen as 0.02 for CDIAC. Sampling time is chosen as 0.001 sec. Simulation results are shown in Figures 5 and 7.

**Figure 6:**
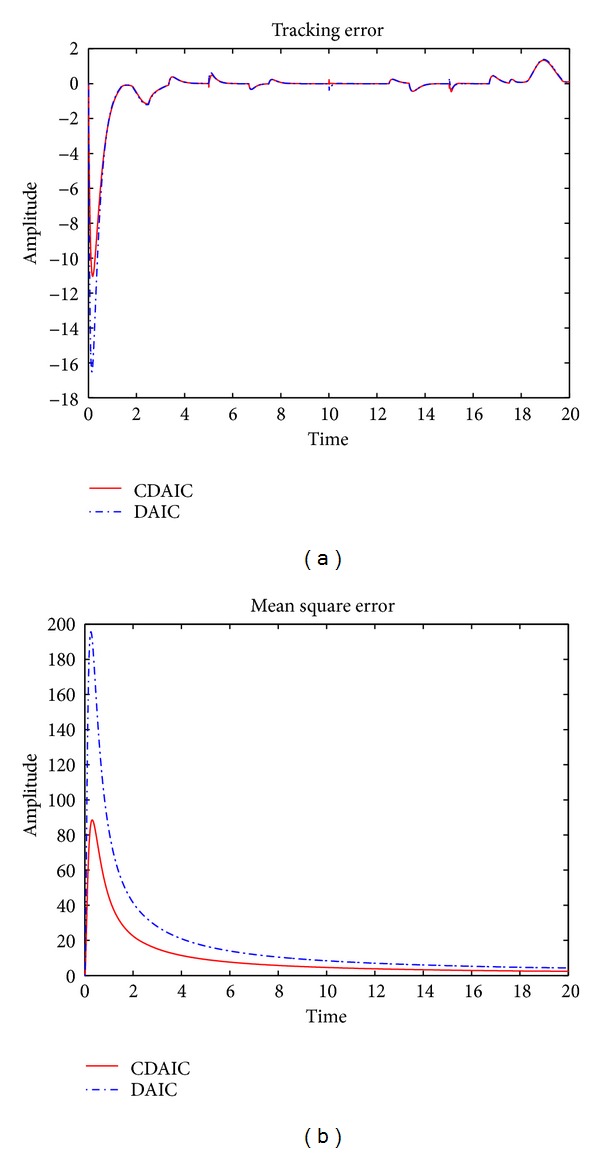
Example 2: simulation results: (a) tracking error; (b) mean square error.

**Figure 7:**
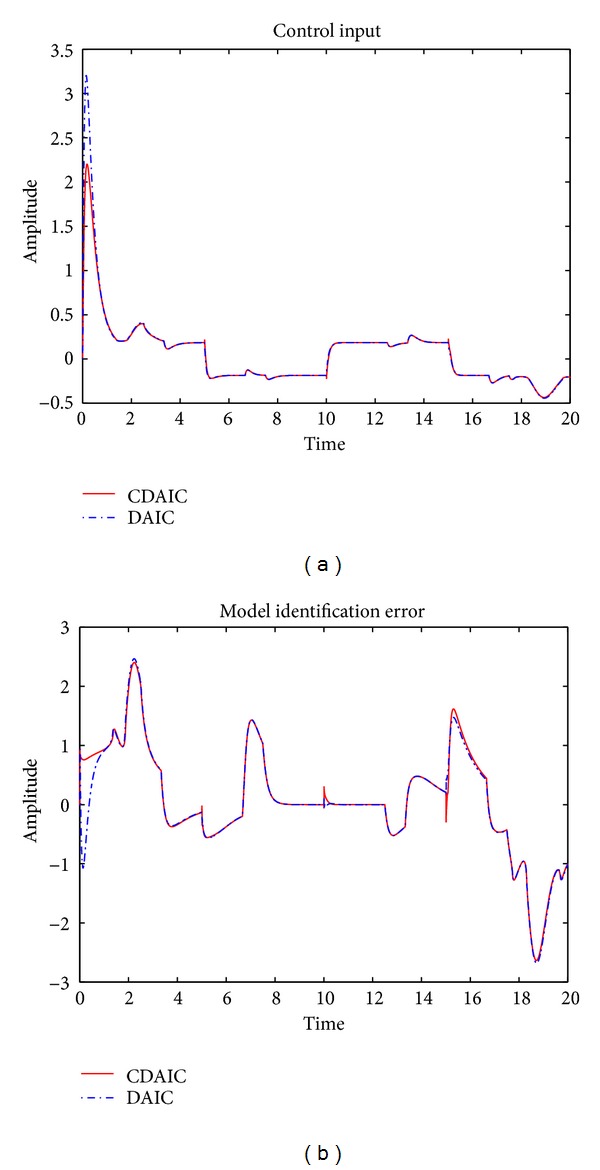
Example 2: simulation results: (a) control input; (b) model identification error.

*n*(*k*), disturbance added to the plant is shown in Figure 5(a). Desired output tracking is shown in Figures 5(b) and 5(c). As shown in Figure 5(c), plant output in CDAIC has less overshoot and converges to desired output quickly compared to DAIC. Tracking error is low for CDAIC compared to DAIC as shown in Figure 6(a). Mean square tracking error of CDAIC is less than DAIC and is depicted in Figure 6(b). Control input and model identification error for both CDAIC and DAIC are shown in Figures 7(a) and 7(b), respectively. Control input in CDAIC is synthesized such that it not only gives better tracking as compared to DAIC but also has good disturbance rejection properties.

## 6. Conclusion

A closed loop direct controller based on NLMS for adaptive tracking of stable plants is proposed. CDAIC is applicable to both minimum and nonminimum phase discrete time linear plants. NLMS algorithm is used for estimation of plant and controller in conjunction with FIR filter at the input stage. Negative feedback has improved the tracking and disturbance rejection properties of DAIC. Simulation results show that CDAIC performs better than DAIC in terms of tracking and mean square error. Little modification can also establish model reference adaptive control (MRAC).
